# 

**Published:** 1875-06

**Authors:** 


### TABLE OF CONTENTS.

ORIGINAL COMMUNICATIONS.

Iridotomy, and its Applicability as a Remedy for Certain Defects of the Eye, with Four Illustrative Cases. By A. W. Calhoun, M. D. : : : 321
Salicine in Diarrhœa and Dysentery. By T. Curtis Smith, M. D. : : 328
Hypodermic Medication. By J. B. Mattison, M. D. : : : : 332
Cases of a Dead Fœtus, Retained in Utero, over Four Months; and Cases of Prolonged Pregnancy. By A. R. Kilpatrick, M. D. : : : 338

SELECTIONS.

A Clinical Lecture on Typhoid Fever and Broncho-Pneumonia. By Francis Delafield, M. D. : : : : : : : : : : 340
Cases Illustrating Different Degrees of Susceptibility to Electricity. By George M. Bread, M. D. : : : : : : : : 344
Early Operation for Hare-lip. By E. R. Maxson, M. D. : : : 352

EXTRACTS AND GLEANINGS.

Jaborandi (Polycarpus Pinnatus)—354. Tincture of Iodine for Cloasma Uterina—355. On the Treatment of Diarrhœa of Typhoid Fever—356. Preventive Midecine—357. Pruritus of the Genitals—358. The Prevention of Sea-Sickness—359 Cinquefoil in Puerperal Peritonitis, and on the Treatment of Diphtheria—360. Diphtheria—361. Proposed Standard for the Doctorate; Apples in Paris—362. Grindelia Robusta in Asthma; Prolapsus Ani Treated by Injections of Ergotine—363. Belladonna in Spasmodic Asthma; Solution of Morphia for Hypodermic Injection—364. Treatment of Pertussis by Inhalation; Extraction of Foreign Bodies from the Eye—365. Studies and Experiments on the Prevention of Contagion in Disease; Death after Delivery, without any Lesions to account for it—366. Passage of a Scissors through the Abdominal Walls after being Swallowed; Life in a Six Months' Fœtus—367. Chloride of Zinc; Is Puerperal Fever Contagious—368. Successful Cæsarean Section in a case of Uterine Fibroids; Compound Tincture of Opium; Tincture of Polk Root—369. Bland on the Treatment of Scarlatina by Hyposulphites and Carbolic Acid; Mustard; Foot-bath in Urticaria; A Simple Method of Removing the Teeth of Children—370. Ablation of the Breast by the Elastic Ligature; Apomorphine; Aromatic Syrup of Senna—371. Aspirator in Retention of the Urine; Standard Weights and Measures; Cure for Warts—372. Dislocation of Humerus after Five Months; Damiana; Chloral and Morphia in Tetanus—373. Hypodermic Medication; Morphia with Atropia for Subcutaneous Administration; Cough and Sweating in Phthisis—374.

EDITORIAL.

Delayed : : : : : : : : : : : : : 375
Inquiry, with Remarks : : : : : : : : : : 376
W. J. M. Gordon : : : : : : : : : : : 378
Ponce de Leon Spring : : : : : : : : : : 379
Communications on hand : : : : : : : : : 380
The State Board of Health; Hymeneal : : : : : : : 381
Dr. S. M. Burnett; Georgia Medical Association; Communications : 382
Ayres' Hernial Truss; Scribner's Illustrated Monthly Magazine; Scientific American; Cash Payments : : : : : : : : : 383

